# Comparison of post-activation performance enhancement (PAPE) after isometric and isotonic exercise on vertical jump performance

**DOI:** 10.1371/journal.pone.0260866

**Published:** 2021-12-02

**Authors:** Salvador Vargas-Molina, Ulises Salgado-Ramírez, Iván Chulvi-Medrano, Leandro Carbone, Sergio Maroto-Izquierdo, Javier Benítez-Porres

**Affiliations:** 1 Exercise Physiology Laboratory, Department of Physical Education and Sports, Faculty of Medicine, University of Malaga, Malaga, Spain; 2 EADE-University of Wales Trinity Saint David, Malaga, Spain; 3 UIRFIDE (Sport Performance and Physical Fitness Research Group), Department of Physical and Sports Education, Faculty of Physical Activity and Sports Sciences, University of Valencia, Valencia, Spain; 4 Faculty of Medicine, University of Salvador, Buenos Aires, Argentina; 5 Department of Health Sciences, European University Miguel de Cervantes, Valladolid, Spain; Universita degli Studi di Milano, ITALY

## Abstract

**Purpose:**

This study aimed to compare the post-activation performance enhancement (PAPE) induced by isometric and isotonic exercise on vertical jump performance.

**Methods:**

18 healthy trained men (25.8±2.7 years; 78.4±8.2 kg; 175.7±6.1 cm; 25.4±1.8 BMI; 126.72±10.8 kg squat 1-RM) volunteered for this study. They randomly performed two different PAPE protocols: Isotonic squats (ISOTS), which consisted of 2 sets of 3 repetitions at 75% of one-maximum repetition (1-RM); and isometric squats (ISOMS), which consisted of 2 sets of 4 seconds of submaximal (75% of 1-RM) isometric contraction at 90°-knee flexion. Countermovement jump (CMJ) height was tested at baseline and 4 minutes after each conditioning set.

**Results:**

CMJ height significantly increased after set 1 in both PAPE protocols (ISOMS: *p* <0.001; ES = 0.34; ISOTS: *p* <0.001; ES = 0.24), with respect to the baseline jump. However, after set 2 no significant changes in CMJ height were observed for any protocol (ISOMS: *p* = 0.162; ES = 0.11; ISOTS: *p* = 0.976; ES = 0.06). No significant differences (p>0.05) were found between both isometric and isotonic exercise conditions.

**Conclusions:**

Despite both protocols showed similar PAPE effects on CMJ height after set 1, none of the protocols demonstrated greater efficacy in increasing subsequent performance in healthy trained men.

## Introduction

Post-activation performance enhancement (PAPE) refers to the acute increase of the explosive neuromuscular capacity, triggered by different types of conditioning activities performed at maximal or near maximal intensity, experienced between 3–10 minutes after the warm-up [1, 2]. PAPE may potentially be associated with increases in neural drive and high threshold motor units activation, and increases in muscle temperature and muscle water content, although its underpinning mechanisms are yet to be defined [3]. Literature has been traditionally focused on the subsequent functional effects of high intensity activities on sports-related tasks, such as sprinting and jumping [4–7]. Indeed, it is widely known that maximal or near-maximal intensities (i.e., intensities above 80% of one-repetition maximum [1-RM]) are needed to induce PAPE in well-trained athletes [8]. Another key factor is resting time between the PAPE protocol and the subsequent activity, due to the PAPE time-course effects (peaking at 7–10 minutes after conditioning activity), specifically for vertical jump performance [9]. Not only exercise intensity or resting time must be considered when designing any given warm-up protocol, but also other several parameters should be considered to get the desired effects, such as, exercise volume, stimuli length, recovery period, and especially, muscle contraction type (i.e., concentric, eccentric or isometric).

However, there is scarce evidence on the different forms of muscle contraction in the achievement of PAPE. The largest body of research has been focused on dynamic isotonic contractions, whereas reduced evidence has been delved into isometrics procedures [3, 10–13]. Nevertheless, it has been shown that isometric contractions possess a lower metabolic cost compared to concentric dynamic contractions [14, 15], which might leads to the assumption that performing maximum isometric contractions during the warm-up may increase subsequent explosive performance while limiting the detrimental effects of accumulated fatigue [16]. However, there are controversial results on the PAPE effects of isometric muscle contractions. Although some investigations have shown greater PAPE effects with the use of dynamic actions that involved the stretch-shortening cycle compared with isometric muscular contractions [17], evidence is not conclusive and some studies have also reported higher potentiation effects in favor of isometric exercise [10, 12, 18].

Moreover, detrimental effects have also been observed when isometric contractions were included in the warm-up [3, 13]. Although this controversy has been attributed to inter-individual variability in response to the conditioning activity [11, 19], it seems appropriated to extend the knowledge about PAPE effects of isometric conditioning activities. So far, only one investigation has documented a comparison of a single set of isotonic and isometric protocols during the squat exercise to promote PAPE on vertical jump performance [12]. Therefore, the aim of this study was to compare the acute effects of applying an isometric activation protocol and an isotonic dynamic one on the countermovement jump (CMJ) performance after one and two sets of the back squat exercise. Findings from the study could be used to specifically select the type of muscle contraction that triggers the greater PAPE on CMJ in healthy trained men. We hypothesize that both protocols will lead to similar PAPE on vertical jump performance, although the isometric protocol will likely induce superior results.

## Methods

### Sample

Eighteen healthy trained men (25.8 ± 2.7 years; 78.4 ± 8.2 kg; 175.7 ± 6.1 cm; 25.4 ± 1.8 BMI; 126.72 ± 10.8 Kg 1-RM squat) volunteered in this study. All of them had, at least, more than two years of resistance training experience, and no history of lower-limb injuries during the last 6 months prior to the study. Participants were requested to avoid any intense lower limb exercise from the 24 hours prior to the start of the familiarization sessions until the completion of the investigation. Participants who reported using doping substances (e.g., anabolic-androgenic steroids) during the last two years and/or who consumed any type of dietary supplement during the program were excluded. Moreover, participants recorded and then maintained their sleeping, eating and drinking habits in the 48 hours prior to each testing session. Additionally, the following restrictions were imposed on volunteers: no food, drinks, or stimulants (e.g., caffeine) to be consumed 3–4 hours prior to each session and no extenuate exercise 24 hours before testing. Participants were informed of the possible harmful risks of the experiment and provided written informed consent agreeing to the conditions of the study. The research protocol was approved by the Ethics Committee of the University of Malaga (code: 38-2019-H) and was conducted in accordance with the guidelines of the Declaration of Helsinki [20].

### Study design

**Fig 1:**
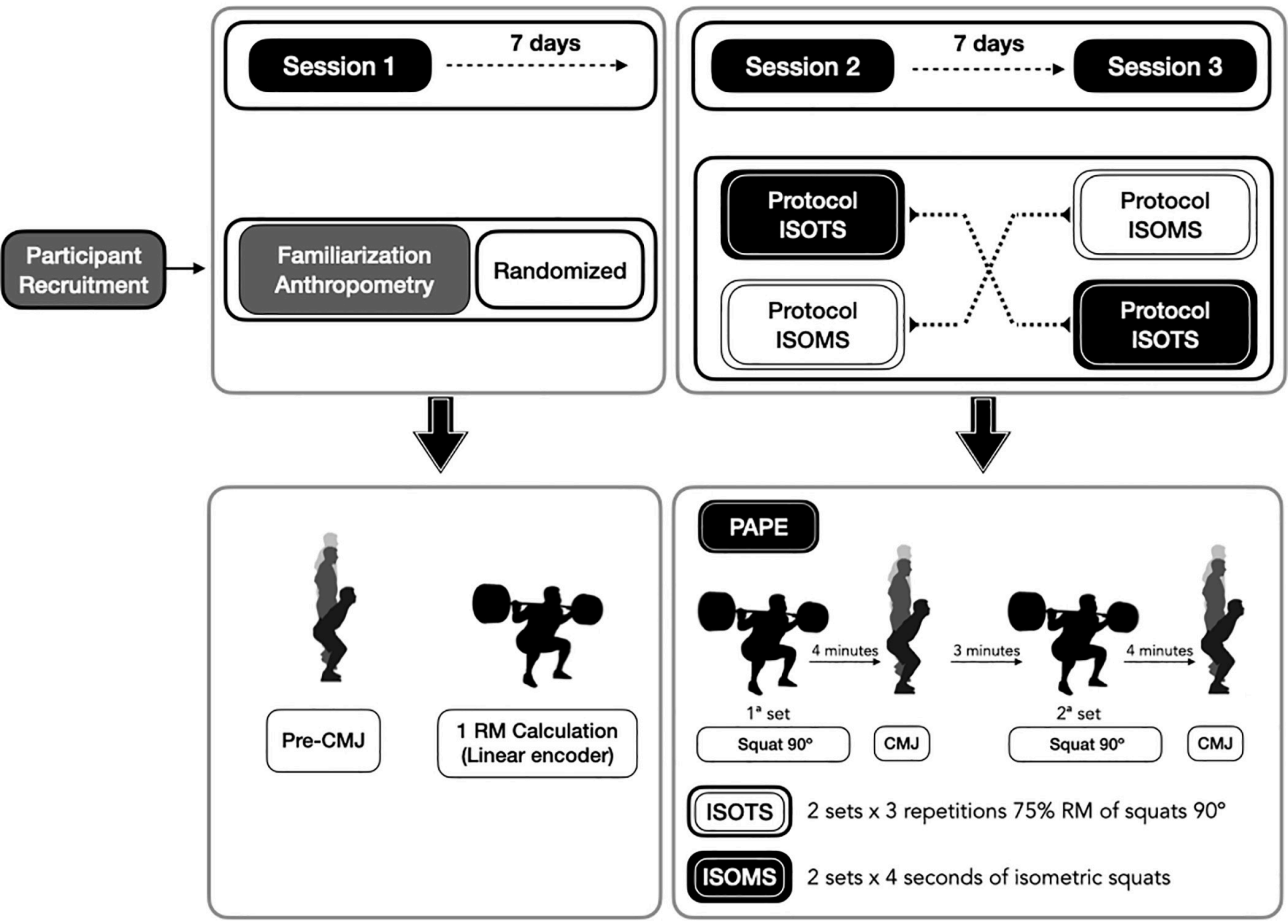
Schematic representation of the study design.

A repeated measures crossover design was used, in which all participants (n = 18) performed a familiarization and baseline assessment session and 2 testing sessions. Following 7 days after familiarization and baseline assessment session, participants were randomly assigned into two different conditions (www.randomizer.org). Half of the participants were randomly assigned to the isotonic dynamic protocol (ISOTS) and the other half to the isometric protocol (ISOMS) to perform session 2. Session 3, in which protocols were exchanged, was performed on the following 7 days (please see Fig 1).

### Procedures

#### Anthropometry

In the first sessions, participants’ height (Seca, Hamburg, Germany) and body mass (Tanita BC-545N, Tokyo, Japan) were recorded. All measurements were performed in the same laboratory and by the same rater. Thereupon, a familiarization session, which consisted of proper instruction on the execution of the CMJ, back squat and 90°-knee flexion isometric squat, was completed.

#### Repetition maximum assessment

Accordingly with previous studies [21], a general warm-up consisting of stationary cycling (BH fitness, Bilbao, Spain) at low intensity for 7–10 minutes, followed by a specific warm-up of a single set of 12–15 repetitions at 40% of the perceived 1-RM was performed. Subsequently, participants performed 2 to 3 sets of 2–3 repetitions at 60–80% of 1-RM. After the warm-up, a progressive incremental test was performed on the back squat exercise using a linear position transducer (LPT, Chronojump-Boscosystem, Barcelona, Spain) to individually estimate the back squat 1-RM. Then, following a previous used protocol [22], the back squat 1-RM was measured. With this purpose, an initial load of 60% of 1-RM was used and 10% increases on every set were applied until reaching a mean propulsive velocity of 0.5 m/s [23], followed by the addition of 5-10kg until 1-RM was achieved. A 3–5 minutes rest interval was interspersed between each attempt. To consider a valid repetition, participants were instructed to reach the 90°-knee flexion. To this end, participants were previously educated in the correct squat technique. An investigator was in charge of guaranteeing the depth of the squat.

#### Countermovement jump (CMJ) height assessment

The CMJ test was performed on a jump mat (Chronojump-Bosco system, Barcelona, Spain) after educating participants on proper execution. They were instructed to initiate the move by reaching 90°-knee flexion, which was standardized using a handheld goniometry and the placement of a cord at the appropriate depth while keeping their hands at the waist and their trunk erected. Participants were requested to perform the movement with no interruption from the beginning to the end of the jump. A total of 3–5 attempts were performed during familiarization before collecting data. After familiarization, two jumps were recorded with a rest interval of 1 min between each attempt. The highest value was computed for further analysis.

#### Conditioning protocols

The second and third experimental sessions began with a specific warm-up that included 5–7 min of stationary cycling, as well as, dynamic joint mobilization. Subsequently, participants completed 2–3 sets of 5 repetitions of the bodyweight squat exercise with 30-seconds rest period between sets followed by 1 set of 3 repetitions of continuous CMJ. After a 3-min rest the baseline CMJ was recorded. Immediately after assessing the baseline CMJ height, participants completed the first set of one of the conditioning protocols (i.e., ISOTS or ISOMS) in a counterbalanced manner. The ISOTS protocol consisted of performing 2 sets of three repetitions at 75% of 1-RM at 90°-knee flexion (where 0° refers to the full knee extension) of the back squat exercise. On the other hand, the ISOMS protocol, consisted of performing 2 sets of 4 seconds of an isometric contraction of the back squat exercise with an external load equivalent to 75% of 1-RM at 90°-knee flexion. In both protocols, after each set a 4-minute recovery period was applied, and immediately after the 4 minutes resting CMJ height was tested following the protocol previously described. Between set 1 and set 2 of each conditioning activity a total of 7 minutes were interpaled. All sessions were supervised by two researchers, that controlled the particular intensity of each exercise, exercise technique and knee flexion during exercise with an electrogoniometer.

### Statistical analysis

Statistical analyses were performed using SPSS v.26.0 (SPSS Inc. Chicago, IL). Results are expressed as mean ± SD. Data distribution was examined for normality using the Shapiro–Wilk test. A mixed-measures analysis of variance (ANOVA) with one between-subjects factor (intervention condition: ISOTS or ISOMS intervention) and one within-subjects factor (time) followed by Bonferroni post-hoc tests was used to investigate differences in CMJ height. The effect size (ES) was calculated for interactions between groups using Hedges’ g. Threshold values for ES were > 0.2 (small), > 0.6 (large) and > 1.2 (very large) [24]. The significance level was set to p < 0.05.

The sample size was estimated using the data from previous studies [19, 25] in which the isotonic and isometric conditioning activities were investigated for the CMJ jump height performance enhancement effect. Based on the effect size of 0.4 for a possible difference in CMJ jump height changes between the pre and post conditions, it was estimated that at least 16 participants were necessary for each condition, with the alpha level of 0.05 and power (1−β) of 0.80 by G*Power (G*Power 3.1.9.2, Heinrich- Heine-Universitat Dusseldorf, Dusseldorf, Germany; http://www.gpower.hhu.de/). Thus, considering possible dropouts and calculation error, 18 participants were recruited.

## Results

**Fig 2:**
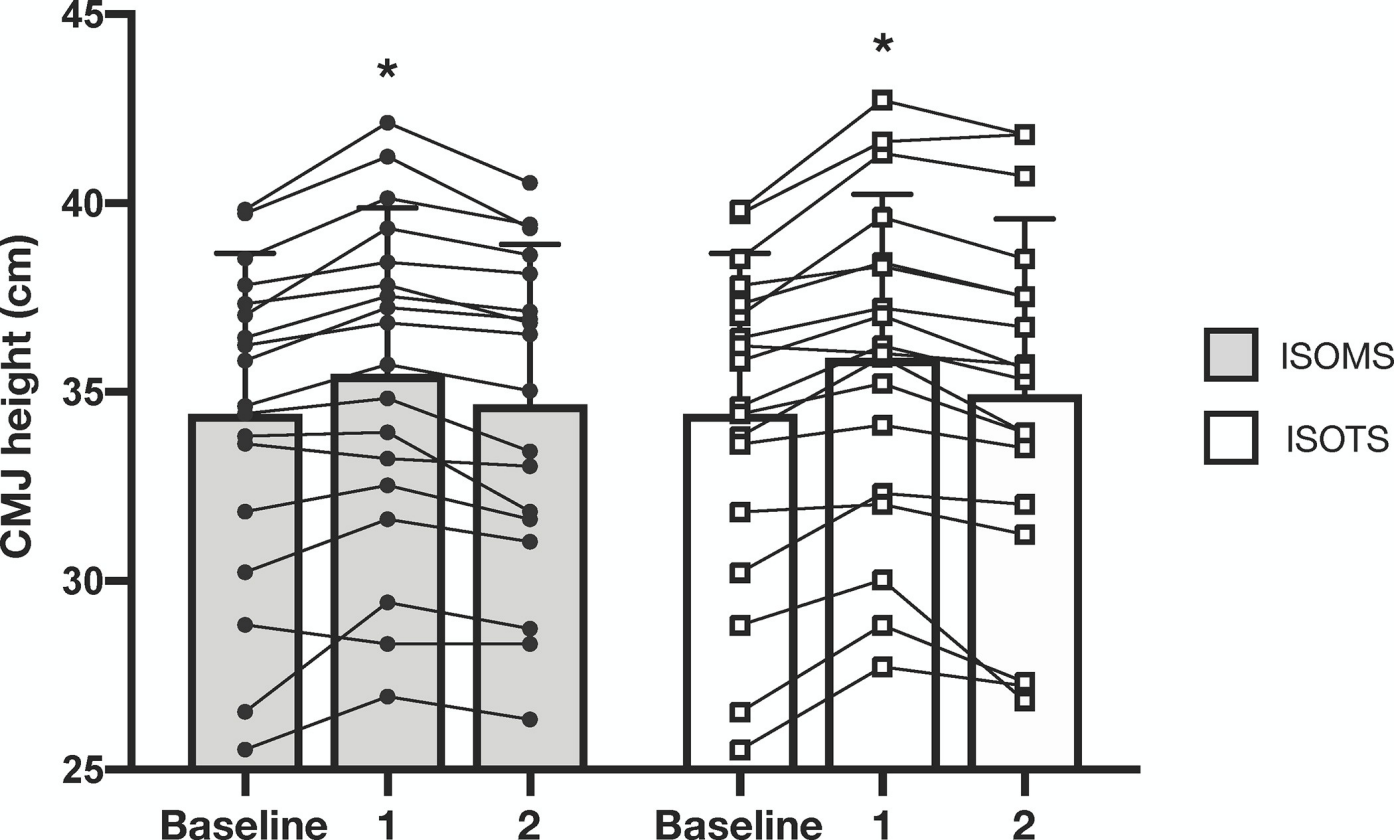
PAPE on CMJ height following isometric and isotonic 90° squat exercise 4 minutes after set 1 (CMJ 1) and 4 minutes after set 2 (CMJ 2). * = meaningful (p<0.001) difference compared to baseline and CMJ 2.

A graphical representation of time effect after isometric or isotonic conditioning activities on CMJ height was reported in Fig 2. Both conditions (i.e., isometric and isotonic) showed significant CMJ height increases 4 minutes after the conditioning activity (i.e., CMJ 1) compared to baseline CMJ height (isometric: p<0.001; F = 36.5; ES = 0.34, 4.3%; isotonic: p<0.001; F = 21.4; ES = 0.24, 3.1%). However, the PAPE effect was dissipated after the second conditioning set, since no differences were found either for ISOMS (p = 0.162; ES = 0.11; 1.5%) nor ISOTS condition (p = 0.976; ES = 0.06; 0.7%) when CMJ 2 was compared with baseline CMJ height. In addition, CMJ height was significant higher after CMJ 1 compared to CMJ 2 for both conditions (ISOMS: p<0.001; ES = 0.22, 2.8%; ISOTS: p<0.001; ES = 0.18, 2.3%). The repeated ANOVA did not report meaningful differences between isometric and isotonic conditioning activities for CMJ vertical jump height.

## Discussion

The aim of this study was to compare the acute effects on vertical jump performance after one set or two sets of isotonic and isometric contractions of the back squat exercise in healthy trained men. PAPE response was achieved after a single set of the selected exercise in both conditions. Therefore, significant vertical jump performance increases were observed in both ISOMS (2.8%) and ISOTS (2.3%) conditions. However, no significant changes were observed after the second set for any condition. Consequently, both dynamic isotonic and isometric contractions added to the warm-up have shown to induce similar PAPE effects on CMJ height when the back squat exercise was performed by well-trained men.

The PAPE phenomenon consists of an acute and transitory increase (peaking at 7–10 minutes after the warm-up) of the neuromuscular capacity induced by a previous contractile activity (i.e., high-intensity strength exercise). PAPE occurs as of 4 minutes after performing the conditioning activity [1]. Unlike post-activation potentiation (PAP, which consists on rate of force development enhancements assessed by an electrically evoked twitch contraction ~3 minutes after the application of an intense voluntary muscular contraction), that underpins potentiation effects through related myosin regulatory light chain phosphorylation mechanisms [1, 26]. PAPE is probably influenced by several other physiological effects, such as increases in muscle temperature, muscle activation and neural coordination, or intracellular water accumulation may also be involve [1, 26]. However, possible PAPE responses may be affected not only by fatigue (for example, high volume doses of strength training exercise or lack of proper resting intervals), but also by perceived loss of coordination in a sequentially performed task, such as a vertical jump [1, 26].

Throughout the scientific literature, subsequent enhancements on explosive performance have been achieved through different strategies [9]. Traditionally, these performance enhancements have been induced by dynamic isotonic exercises based on the individual concentric maximum strength capacity. Indeed, PAPE response may be influenced by the volume and intensity of the conditioning activity as well as the rest period between the warm-up and the subsequent activity (i.e., depending on the net balance between fatigue and performance enhancement, which co-exist at varying degrees after the warm-up) [8, 27]. Seitz & Haff [8] have shown that traditional high-intensity resistance exercises produced considerably larger PAPE effects than traditional moderate-intensity and maximal isometric conditioning activities. Although these performance enhancements were highly individualized and largely dependent of the level of strength and experience (stronger individuals are able to exhibit a greater effect than their weaker counterparts), exercise mode (half squat showed greater effects than deeper squat), type of load (high-intensity maximal loads produces greater levels of PAPE than a sub-maximal load performed at a given percentage of 1-RM), rest period between the conditioning activity and subsequent exercise (the greatest effect is realized after 5–7 minutes recovery intervals between the warm-up and the subsequent exercise), and balance between volume and intensity. Hence, it seems that athletes and highly-experienced practitioners may be more benefited by single-set and high-intensity strengthening activities during the warm-up [28].

However, the strategies that have been used to trigger PAPE and enhance vertical jump performance employing isometric contractions have not been yet investigated in detail. In this regard, it is well-known that the acute responses provided by isometric muscle contractions generate a lower metabolic cost and reduced fatigue compared with isotonic concentric muscle contractions [12]. For such reason, isometric exercise has been proposed as a valid option to promote subsequent performance enhancements on explosive movements such as sprinting [19] or rowing [17, 29] in well-trained athletes. However, the relationship between the application of isometric contractions and the effect on acute jumping performance is not well documented. Consequently, it is difficult to establish an application protocol and to have a perspective due to the methodological heterogeneity found. For example, it has been found that 3 isometric contractions of 3 seconds at 50–90% of the maximal voluntary isometric contraction (MVIC) in the 140°-knee flexion squat exercise promoted significant increases in CMJ height 12 minutes after the warm-up [25]. These results are in line with our results. Notwithstanding, isometric conditioning activities have also led to detrimental effects. A similar isometric protocol but using the knee extension exercise did not register any improvement in the CMJ height [3]. This implies that the highest similarity (i.e., kinetic and kinematic demands and gesture similarity) between the warm-up conditioning activity and the evaluated task, the largest enhancements in subsequent task-specific performance [25]. Indeed, participants were instructed to perform the back squat exercise and the CMJ movement with a high resemblance. Moreover, isometric exercise is highly prescribed at maximal or near-maximal intensity and with a high volume (i.e., time under tension). This may increase accumulated fatigue [30], and a reduction on the peak concentric power or peak torque in lower limbs, as has been previously shown [13]. However, it was not observed in our study likely due to the submaximal intensity of the ISOMS protocol (i.e., 75% of 1-RM). Moreover, it has been reported that isometric muscle contractions generate less muscle damage and soreness compared to dynamic actions (especially eccentric exercise) [31]. For such reason, isometric exercise may have potential applications in sports performance and rehabilitation settings.

In addition, studies comparing the effects of isometric and isotonic conditioning activities on subsequent performance are scarce. Rixon et al. [12] compared the subsequent explosive enhancement after a single set of dynamic and isometric squat exercise. They compared the effects on vertical jump performance of a single set of 3 repetitions at 90% of 1-RM, and a single set of 3 repetitions of 3 seconds of maximum isometric muscle contraction [12]. The isometric protocol found superior gains in CMJ height in both men and women (2.9% and 1.7%, respectively) compared to the isotonic protocol (1.2% and -1.5%, respectively) [12]. However, these results should be considered with caution since both protocols were not randomized nor counterbalanced. Additionally, there are some differences with respect to this study, such as time under tension of the isometric contraction. In this study, a total of 4 seconds of isometric muscle contraction were prescribed compared to the total 9 seconds in the aforementioned study. Although, it seems that it is not necessary to perform maximal efforts [3], such marked improvements were not found in the ISOMS protocol, likely due to the lower time under tension compared to Rixon’s et al. [12] study. Exercise intensity may be another key factor that explains the contradictory effects. Likely, to achieve optimal PAPE effects using an isometric exercise, exercise intensity should be maximal or near maximal [12, 32]. However, lesser time under tension application (i.e., short and concentrated isometric actions performed at functional angles with intensities above the 1-RM) should be considered, as proposed by Berning et al. [33]. Thus, optimizing the PAPE responses (longer and higher acute performance enhancements) by reaching reserve motor units [32] and favoring greater excitability and recruitment of fast-twitch muscle fibers [3, 12, 32, 34]. Which in turn, it would also explain the lack of PAPE after the second set in the ISOMS condition. Even though, more research is warren to define the time course effects of isometric contractions activities.

### Limitations

This study has some limitations and flaws that are important to mention. The fact that all the participants were highly trained subjects might influenced our results [10]. In addition, the kinetic variables of the jump have not been recorded, which could have provided a greater number of outcomes that allowed us to better understand the results of our research in a greater extent. Similarly, electromyographic analysis or any other neurophysiological parameter would have allowed us to deepen in the underpinning PAPE mechanisms, and therefore it is also a limitation of this study. Moreover, this study possesses a lack of compliance with the study design considerations for PAPE studies proposed by Blazevich & Babalult in 2/9 items [1]. Since no control of muscle temperature was performed nor researchers were not blinded. It also should be noted, that the 90°-knee flexion determination and standardization during the CMJ test could influence the results, since it has been shown that CMJ depth variability can affect vertical jump performance [35]. Finally, another limitation of this study was not including a third condition to serve as a control which would have allowed us to verify that the changes in CMJ performance were uniquely attributable to the conditioning activities and not just to the warm-up.

## Conclusions

It can be concluded that post-activation performance enhancements on CMJ height can be achieved with both dynamic and isometric strategies within the 90°-knee flexion back squat exercise in well-trained men. A single set of both ISOMS and ISOTS conditions enhanced vertical jump height 4 minutes after the warm-up, but a second set performed 7 minutes later led to detrimental effects. Type of muscle contraction of the selected conditioning activities should be considered when designing a warm-up protocol. ISOMS may promote similar or even greater improvements in subsequent explosive performance with lower metabolic cost and exercise-induced muscle damage compared to isotonic dynamic exercises. However, further investigation is warren to provide specific recommendations for practical application of isometric PAPE protocols.
